# Molecular mechanism of autophagy and apoptosis in endometriosis: Current understanding and future research directions

**DOI:** 10.1002/rmb2.12577

**Published:** 2024-04-20

**Authors:** Hiroshi Kobayashi, Shogo Imanaka, Chiharu Yoshimoto, Sho Matsubara, Hiroshi Shigetomi

**Affiliations:** ^1^ Department of Gynecology and Reproductive Medicine Ms.Clinic MayOne Kashihara Japan; ^2^ Department of Obstetrics and Gynecology Nara Medical University Kashihara Japan; ^3^ Department of Obstetrics and Gynecology Nara Prefecture General Medical Center Nara Japan; ^4^ Department of Medicine Kei Oushin Clinic Nishinomiya Japan; ^5^ Department of Gynecology and Reproductive Medicine Aska Ladies Clinic Nara Japan

**Keywords:** apoptosis, autophagy, endometriosis, mitochondria, mitophagy

## Abstract

**Background:**

Endometriosis is a common gynecological condition, with symptoms including pain and infertility. Regurgitated endometrial cells into the peritoneal cavity encounter hypoxia and nutrient starvation. Endometriotic cells have evolved various adaptive mechanisms to survive in this inevitable condition. These adaptations include escape from apoptosis. Autophagy, a self‐degradation system, controls apoptosis during stress conditions. However, to date, the mechanisms regulating the interplay between autophagy and apoptosis are still poorly understood. In this review, we summarize the current understanding of the molecular characteristics of autophagy in endometriosis and discuss future therapeutic challenges.

**Methods:**

A search of PubMed and Google Scholar databases were used to identify relevant studies for this narrative literature review.

**Results:**

Autophagy may be dynamically regulated through various intrinsic (e.g., PI3K/AKT/mTOR signal transduction network) and extrinsic (e.g., hypoxia and iron‐mediated oxidative stress) pathways, contributing to the development and progression of endometriosis. Upregulation of mTOR expression suppresses apoptosis via inhibiting the autophagy pathway, whereas hypoxia or excess iron often inhibits apoptosis via promoting autophagy.

**Conclusion:**

Endometriotic cells may have acquired antiapoptotic mechanisms through unique intrinsic and extrinsic autophagy pathways to survive in changing environments.

## 1 INTRODUCTION

Endometriosis is a common estrogen‐dependent gynecological disease that causes pelvic pain, dysmenorrhea, and infertility.^1^ It affects 10% of women during their reproductive period. Endometriosis is characterized by abnormal growth of endometrium‐like glandular and stromal cells outside of the uterine cavity, usually in the peritoneum, ovaries, and pelvic cavity. The most commonly accepted theory is the retrograde menstrual reflux (i.e., Sampson's hypothesis).^2^ The normal endometrium retrogrades into the peritoneal cavity and is exposed to severe hypoxic stress.^3^, ^4^ How regurgitated endometrial tissues survive, implant, and grow as endometriotic lesions under harsh conditions is not well understood. Endometriosis is characterized by enhanced proliferation and diminished apoptosis of endometrial and stromal cells.^4^ Apoptosis has been shown to be regulated by autophagy through several mediators.^5^ The difference between autophagy and apoptosis can be summarized in two words: “self‐eating” and “self‐killing,” and autophagy is an efficient regulator of apoptosis.^6^ Autophagy in eukaryotic cells promotes the degradation of subcellular elements to maintain cellular homeostasis via the autophagosome (double‐membrane vesicles)‐lysosome system.^5^, ^7^ Intracellular materials degraded by autophagy contain the cargo composed of cytoplasmic components including mitochondria, lipids, oxidized proteins, macromolecules, abnormal protein aggregates, and damaged or aged organelles.^5^, ^8^ This process induces an alternative source of bioenergetic metabolites and recycles nutrients for survival and cellular protection in response to environmental stress.^3^, ^9^ For example, it is well accepted that autophagy has been linked to a range of physiological and pathological processes, including reproduction, embryo implantation,^10^ myopathy, metabolic disorder, neurodegenerative disease,^11^, ^12^ cardiovascular disease, and cancer.^7^, ^9^, ^13^

Autophagy is classified into different categories: macroautophagy, selective autophagy, chaperone‐mediated autophagy, and microautophagy.^14^ Macroautophagy consists of several steps: initiation, induction, phagophore elongation, autophagosome formation and maturation, autolysosome formation, and proteolytic degradation of the contents.^14^, ^15^ Selective autophagy is the elimination of specific cellular components such as mitochondria (i.e., mitophagy) and requires recognition of injured mitochondria and subsequent activation of autophagy.^14^ Mitochondria of eukaryotic cells stem from a bacterium 1.5 billion years ago, regulate oxidative phosphorylation, and maintain cellular functions.^16^ Until now, we have been elucidating the molecular and cellular mechanisms underlying endometriosis pathogenesis, focusing on energy metabolism, mitochondrial dynamics, and cellular redox homeostasis.^17^ Mitochondria supply adenosine triphosphate (ATP) from aerobic respiration and orchestrate cell proliferation and development, but they also form reactive oxygen species (ROS) as by‐products in the electron transport chain.^16^, ^17^ Increased ROS production causes impairment of mitochondria, so mitophagy has evolved to eliminate the dysfunctional mitochondria.^16^, ^18^ Thus, mitochondria have evolved mechanisms for quality control and cellular homeostasis,^19^ but they also serve as a hub for the apoptosis signaling pathways.^20^ This is because mitophagy and apoptosis are often induced in response to common stimuli (e.g., mitochondrial dysfunction, oxidative stress, and calcium ion concentration^11^).^20^, ^21^ Although autophagy/mitophagy and apoptosis are closely related to each other and share some common signals, they are distinct processes.^21^ The signal transmission between autophagy/mitophagy and apoptosis in endometriosis is complex and still not fully understood.^22^ Importantly, autophagy and mitophagy exert opposite functions, protective and lethal, so they can inhibit or promote apoptosis.^21^, ^23^ Several intrinsic (e.g., genetic predisposition, certain signal transduction pathways, female hormonal stimuli, ATP content, and p53 status^24^) and extrinsic factors (e.g., hypoxia, oxidative stress, iron concentration, reduced nutrient supply, metabolic stress, and endoplasmic reticulum stress^25^, ^26^, ^27^) have been reported to be involved in the regulation of autophagy/mitophagy in endometriotic tissues.^5^, ^28^ Endometriosis cells are thought to control apoptosis by activating or suppressing autophagy/mitophagy in response to both stimuli. In this review, we summarize our current understanding of how autophagy/mitophagy and apoptosis modulate endometriosis development and progression and discuss future directions for research.

## 2 MATERIALS AND METHODS

### 2.1 Search strategy and selection criteria

We conducted a narrative review of the literature that focuses on autophagic, mitophagic, and apoptotic function in endometriosis. Electronic databases including PubMed and Google Scholar were searched for literature published up to the October 31, 2023, combining the following keywords: “Apoptosis,” “Autophagy,” “Endometriosis,” “Mitochondria,” and “Mitophagy.” The search strategy using the keyword string and combination of Boolean operators is shown in Table 1.

**TABLE 1:** The search strategy.

Search mode	The keyword and search term combinations
Search term 1	Endometriosis OR Pelvic endometriosis OR Endometrioma OR Deep infiltrating endometriosis
Search term 2	Mitochondria OR
Search term 3	Autophagy OR Autophagic
Search term 4	Mitophagy OR Mitophagic
Search term 5	Apoptosis OR Cell death
Search	Search term 1 AND Search term 2
	Search term 1 AND Search term 3
	Search term 1 AND Search term 4
	Search term 1 AND Search term 5
	Search term 1 AND Search term 2 AND Search term 3
	Search term 1 AND Search term 2 AND Search term 4
	Search term 1 AND Search term 2 AND Search term 5
	Search term 1 AND Search term 2 AND Search term 3 AND Search term 4 AND Search term 5

Papers reporting patients' data and in vitro and animal studies conducted to investigate the potential effect and underlying molecular mechanism were also included.

## 3 THE REGULATORY MECHANISMS OF AUTOPHAGY

**FIGURE 1:**
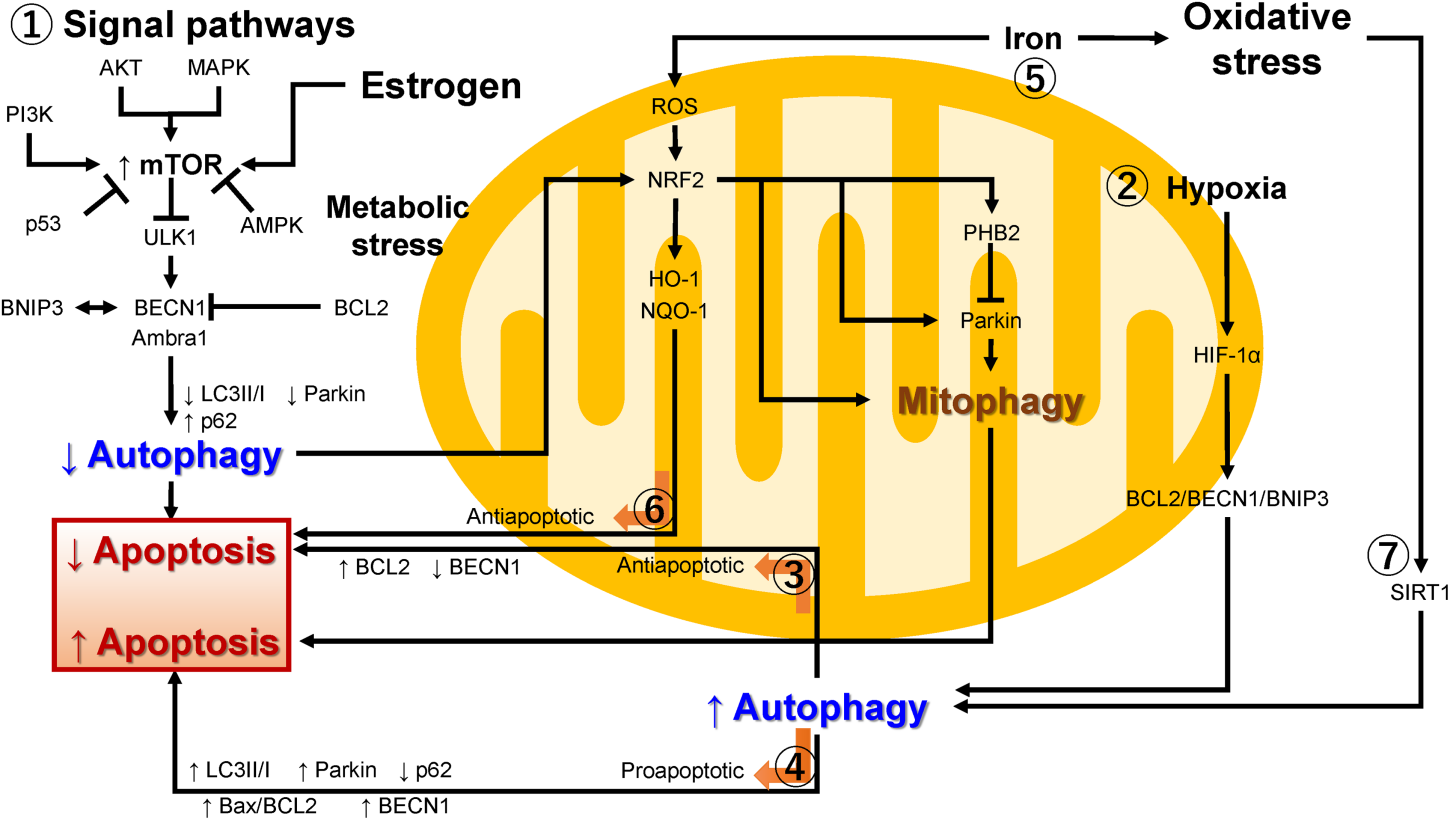
A conceptual diagram of current understanding of signaling pathways that control autophagy, mitophagy, and apoptosis in endometriosis. Route 1, The interplay between autophagy and apoptosis through shared signaling pathways (e.g., PI3K/AKT/mTOR pathway, estrogen signals, AMPK, or p53); Route 2, Induction of HIF‐1α stabilization by hypoxia; Route 3, Suppression of apoptosis by upregulation of Bcl‐2 expression; Route 4, Apoptosis induced by excessive autophagy; Route 5, Autophagy regulated by iron overload; Route 6, Suppression of apoptosis by upregulation of antioxidant gene expression; and Route 7, Regulation of autophagy by SIRT1‐dependent deacetylation.

Essentially, autophagy exerts its protective effects by adapting to stress conditions and creates a favorable environment for tissue repair and survival.^3^, ^9^ For example, under conditions of nutrient starvation or hypoxia, autophagy acts as a prosurvival mechanism. Moderate autophagy can attenuate apoptosis by removing damaged constituents and depolarized/impaired mitochondria in both physiological and pathological conditions.^26^, ^27^, ^28^ Cells can cope with unfavorable environments and cellular damage, such as mitochondrial stress, through autophagy, until a time at which the damage done becomes too great.^29^, ^30^ However, overactivated autophagy beyond a certain threshold level (i.e., excessive autophagy) usually leads to cell death through the cytochrome c release from mitochondria and subsequent caspase activation.^29^, ^30^ For a comprehensive review of the autophagic pathway, refer to Feng et al.^31^ The authors summarized recent advances in the core molecular machinery of autophagy, focusing on the structural information. We provide a conceptual diagram of current understanding of signaling pathways that control autophagy, mitophagy, and apoptosis in the pathogenesis of endometriosis. The autophagy process is associated with multiple cellular signaling transduction pathways (e.g., phosphatidylinositol 3‐kinase (PI3K)/AKT/mammalian target of rapamycin (mTOR) signaling^32^ and hypoxia‐inducible factor (HIF)‐dependent pathways^25^) and downstream target molecules such as autophagy‐related gene (ATG) proteins. This section mainly provides an overview of the PI3K/Akt/mTOR signal transduction pathway (Figure 1, ①), and other extrinsic/environmental factors are summarized in the Section 5. MTOR, a serine/threonine kinase, is a negative regulator of autophagy induction; mTOR pathway activators (e.g., via PI3K, Akt, and mitogen‐activated protein kinases (MAPK) signalings) suppress autophagy, whereas mTOR pathway inhibitors (e.g., via AMPK and p53 signalings) promote autophagy (Figure 1, ①).^33^ More than 30 ATG proteins, downstream targets of mTOR, are involved in autophagy.^34^ MTOR suppresses the function of the downstream target molecule, serine/threonine kinase UNC‐51‐like kinase 1 (ULK1).^5^, ^31^ Moreover, ULK1 located in autophagosomes phosphorylates and activates Beclin1 (BECN1, also known as ATG6) and BECN1‐regulated autophagy 1 (Ambra1), interacts with Ambra1, and promotes autophagosome nucleation.^5^, ^31^ The antiapoptotic proteins (e.g., BCL2 apoptosis regulator (Bcl‐2), Bcl‐XL, and Bcl‐B) bind to Beclin1, prevent its association with the PI3K Class III complex, and inhibits the Beclin1‐mediated autophagy.^35^, ^36^ Therefore, Bcl‐2 not only downregulates apoptosis but also prevents autophagy through its inhibitory interaction with Beclin1.^21^, ^31^ In other words, Bcl‐2 binding to Beclin1 inhibits autophagy.^36^ Additionally, BCL2 and adenovirus E1B 19 kDa‐interacting protein 3 (BNIP3) is a protein homologous to Bcl‐2, interacts with Bcl‐2, and modifies the function of Beclin1.^37^ We discuss the interaction between Bcl‐2, Beclin1, and Bnip3 later (see Subsection 4.2). Furthermore, autophagosome formation requires a unique protein‐lipid conjugate, a complex of multiple ATG proteins and microtubule‐associated protein light chain 3 (LC3).^5^, ^31^, ^38^ LC3 is converted from LC3‐I (i.e., LC3 bound to phosphatidylethanolamine) to LC3‐II (i.e., LC3 specifically associated with autophagosome membranes), which promotes autophagosome nucleation and formation.^38^, ^39^ LC3‐II is also involved in fusion with lysosomes and generation of autolysosomes. p62/Sequestosome 1 (SQSTM1) interacts with LC3 on autophagosomes and is degraded through fusion with lysosomes.^38^ Therefore, autophagy activation is characterized by increased LC3‐II and reduced p62.^38^

## 4 THE REGULATORY MECHANISMS OF MITOPHAGY

**FIGURE 2:**
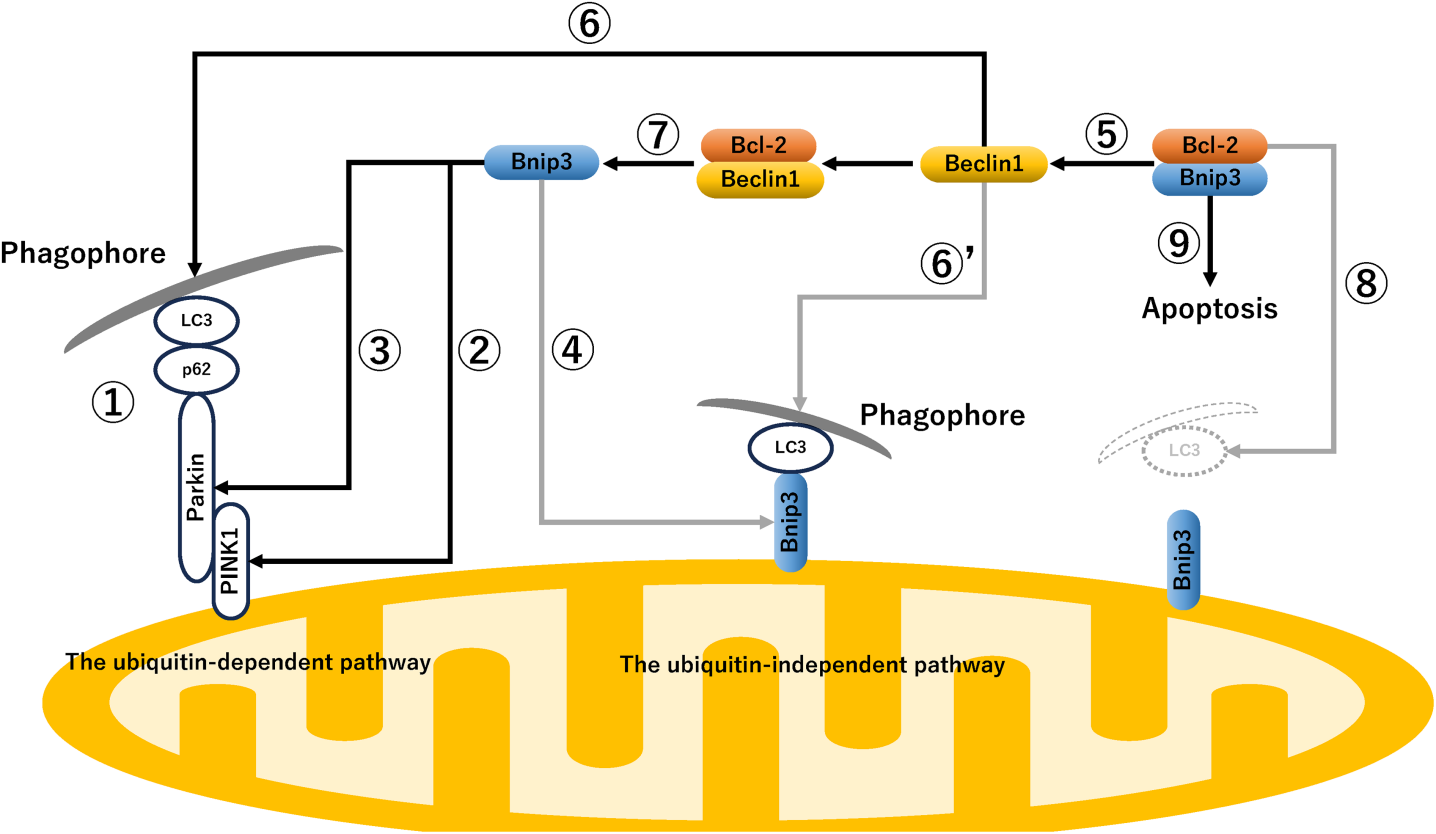
An overview of the molecular mechanism of mitophagy in endometriosis. Route 1, ubiquitin‐dependent mitophagy pathway; Route 2, Inhibition of PINK1 degradation by Bnip3; Route 3, Parkin translocation to mitochondria by Bnip3; Route 4, The ubiquitin‐independent pathway orchestrated by a receptor protein, BNIP3; Route 5, The interrelationship between molecules, Bcl‐2, Bnip3, and Beclin1: Bnip3 competes with Beclin1 for the binding site of Bcl‐2 protein and releases Beclin1; Route 6, Beclin1 is involved in autophagosome elongation, maturation, and autophagolysosome formation in the ubiquitin‐dependent pathway; Route 6′, Beclin1 is involved in autophagosome elongation, maturation, and autophagolysosome formation in the ubiquitin‐independent pathway; Route 7, The interrelationship between molecules, Bcl‐2, Bnip3, and Beclin1: Beclin1 competes with Bnip3 for the binding site of Bcl‐2 protein and releases Bnip3; Route 8, Excessive Bcl‐2 downregulates mitophagy through binding to Bnip3; and Route 9, Apoptosis induction by Bnip3 via inhibition of Bcl‐2. The pathways indicated by the black arrow has been proven to contribute to the process of mitophagy in endometriosis. The gray arrow indicates that despite the demonstrated effects of mitophagy on some other cells, there is no direct evidence for this reasoning in endometriotic cells.

Increasing attention is now being paid to the deregulation of mitochondrial dynamics in endometriosis.^17^, ^40^, ^41^ The maintenance of mitochondrial biogenesis and homeostasis is achieved by mitochondrial quantity and quality control through continual fusion and fission, i.e., mitochondrial dynamics.^40^, ^41^ Mitochondrial fusion is driven by the mitochondrial outer membrane dynamin like GTPase fusion proteins, mitofusins 1 and 2 (MFN1 and MFN2), and the mitochondrial inner membrane dynamin like GTPase fusion protein, optic atrophy 1 (OPA1).^42^ Enhanced mitochondrial fusion facilitates mitochondrial genomic repair and promotes oxidative phosphorylation and generation of ATP, allowing cells to survive even under stressful environments such as starvation.^41^ On the other hand, eukaryotic cells have evolved mechanisms to maintain cell survival by eliminating dysfunctional mitochondria themselves due to enhanced production of ROS, decreased rates of oxidative phosphorylation, depletion of cell ATP pool, and increased numbers of mitochondrial DNA mutations.^41^ Mitochondrial fission is crucial for mitochondrial quality control mechanism that eliminates damaged mitochondria via mitophagy.^40^, ^41^ Mitochondrial fission is primarily mediated by the large GTPase dynamin‐related protein 1 (DRP1).^43^ Regulation of the homeostatic balance between mitochondrial fusion and fission is critical for determining mitophagy‐mediated cell survival and death. Impaired mitochondrial dynamics or dysfunctional mitophagy is known to be associated with many diseases, including neurodegenerative disorders,^44^ metabolic disorders,^45^ cancer,^46^ and endometriosis.^17^ Therefore, a detailed understanding of the molecular mechanism and function underlying mitophagy is crucial for elucidating the pathogenesis of endometriosis. Next, Figure 2 provides an overview of the molecular mechanism of mitophagy. Mitophagy is regulated by the ubiquitin‐dependent and ‐independent pathways.^47^

### 4.1 Ubiquitin‐dependent mitophagy pathway

We summarize ubiquitin‐dependent mitophagy in endometriosis.^15^, ^48^, ^49^, ^50^, ^51^ Ubiquitin‐dependent mitophagy is regulated by phosphatase and tensin homolog (PTEN)‐induced putative kinase 1 (PINK1) and E3 ubiquitin ligase Parkin (PRKN) (Figure 2, ①).^52^, ^53^ Once mitochondria are damaged, stabilization of PINK1 promotes mitochondrial recruitment of Parkin and ubiquitin, which in turn further accumulates autophagy adapter proteins (e.g., p62 and Ambra1) and LC3.^54^, ^55^, ^56^ Phagophores expand, sequester portions of the organelles, close, and create autophagosomes. Finally, fusion of autophagosomes and lysosomes generates autolysosomes where impaired mitochondria and p62 are degraded. Bnip3 inhibits PINK1 degradation and promotes its accumulation in the outer mitochondrial membrane (Figure 2, ②)^54^, ^56^ and also promotes Parkin translocation to mitochondria (Figure 2, ③).^57^ In fact, ubiquitin‐dependent (i.e., Parkin‐required) mitophagy has been shown to regulate endometriosis apoptosis via upregulation of macrophage stimulating 1 (Mst1)^51^ or Prohibitin2 (PHB2)^48^ (see Figure 1). This suggests that mitophagy regulates apoptosis during endometriosis development.

### 4.2 Ubiquitin‐independent mitophagy pathway (receptor‐dependent mitophagy pathway)

In contrast, the ubiquitin‐independent pathway is mediated by tail‐anchored proteins in the outer mitochondrial membrane.^18^, ^47^ These mitophagy receptors consist of at least five proteins, including BNIP3, BCL2 interacting protein 3 like (BNIP3L), FUN14 domain containing 1 (FUNDC1), BCL2 like 13 (BCL2L13), FKBP prolyl isomerase 8 (FKBP8).^47^, ^56^, ^58^, ^59^ Among these mitophagy receptors, the function of Bnip3 is shown in Figure 2. Bnip3 interacts with LC3, linking the phagophore to the targeted mitochondria to mediate mitophagy^55^, ^60^ (Figure 2, ④). Moreover, coordination between autophagy and apoptosis is regulated by Bcl‐2 family proteins.^21^ Both Beclin1 and Bnip3 interact with Bcl‐2 in a mutually exclusive manner.^36^, ^61^ Bnip3 competes with Beclin1 for the binding site of Bcl‐2 protein and releases Beclin1^55^ (Figure 2, ⑤). Beclin1 forms a high‐affinity complex with Atg14, Ambra1, p150, and PI3K Class III and is involved in autophagosome elongation, maturation, fusion with lysosomes, and autophagolysosome formation, which progresses mitophagy^55^ (Figure 2, ⑥, ⑥’). Since Beclin1 and Bnip3 compete for the same binding sites in the Bcl‐2 molecule (Figure 2, ⑦), Beclin1 promotes the Bnip3‐mediated mitophagy^62^ (Figure 2, ②, ③, and ④). Additionally, Bnip3 disrupts interaction between the Beclin1 and Bcl‐2 proteins to promote the Beclin1‐mediated autophagy^61^ (Figure 1, ①). Excessive Bcl‐2 protein downregulates autophagy and mitophagy through binding to the autophagy modulators Beclin1 (Figure 1, ①) and Bnip3^35^, ^36^ (Figure 2, ⑧). Furthermore, Bcl‐2 family proteins suppress PINK1‐PRKN‐dependent mitophagy through inhibiting the Bnip3‐mediated autophagy^63^ (Figure 1, ①). On the other hand, Bnip3 triggers apoptosis through suppressing Bcl‐2 function and promoting BCL2 associated X apoptosis regulator (Bax)‐dependent apoptosis^64^ (Figure 2, ⑨). Therefore, Bnip3 is known as a dual‐function regulator of apoptosis and autophagy/mitophagy.^37^ In addition, Bcl‐2 exerts antiapoptotic and antiautophagic functions. Collectively, the balance between Bcl‐2 and Beclin1/Bnip3 largely affects mitophagy. These proteins serve as important mediators that mediate coordination between autophagy/mitophagy and apoptosis.^63^ Although there are several reports on the role of autophagy‐related markers Beclin‐1 and LC3 in endometriosis,^65^, ^66^, ^67^, ^68^, ^69^, ^70^, ^71^ only a few studies have exploited in vitro and animal models to study the function of the Bnip3 molecule.^15^, ^49^ Evidence in endometriosis is still scarce regarding the pathways ④, ⑥’, and ⑧ (Figure 2).

## 5 MECHANISM OF INTERACTION BETWEEN AUTOPHAGY/MITOPHAGY AND APOPTOSIS IN ENDOMETRIOSIS

In this section, we summarize how autophagy and mitophagy control apoptosis and the underlying molecular mechanisms, focusing on the intrinsic (Subsections 5.2 and 5.3) and extrinsic (Subsections 5.1, 5.4, 5.5, 5.6, and 5.7) pathways in endometriosis. Studies have shown that autophagy is associated with the regulation of menstruation and the pathogenesis of endometriosis.^7^ The autophagy level in the secretory phase was significantly higher than that in the proliferative phase in the stromal cells of the normal endometrium.^72^, ^73^ However, the ectopic endometrial epithelial and stromal cells had markedly lower autophagy levels during the proliferative and secretory phases compared with the normal endometrium.^66^, ^73^, ^74^, ^75^ Also, the autophagy levels quantitatively differed among distinct endometriotic lesions (ovaries, fallopian tubes, peritoneal, gastrointestinal, and skin).^75^ Normal endometrial cells with reduced autophagic function may reflux into the peritoneal cavity and proliferate as ectopic endometriotic cells by restricting apoptosis.^4^, ^5^, ^7^, ^74^ In contrast, other researchers have reported upregulation of autophagy levels in the ectopic endometrium of patients with ovarian endometriosis.^76^, ^77^ Upregulation of Beclin1 and LC3II expression and downregulation of p62 expression were detected in tissue samples and endometriotic cells from patients with endometriosis.^76^, ^77^ Therefore, previous studies have yielded mixed or inconsistent results regarding the levels of autophagy in endometriosis. To explore reasons for inconsistent reports, we then focus on how various types of stressors (e.g., signal transduction, oxidative stress, hypoxia, or energy starvation) regulate autophagy‐mediated apoptosis in endometriotic cells.

### 5.1 PI3K/AKT/mTOR pathway

As mentioned in section 3, the PI3K/AKT/mTOR signaling pathway is a major pathway involved in the initiation and regulation of autophagy,^78^, ^79^, ^80^, ^81^, ^82^ and upregulation of PI3K and Akt expression impairs autophagy in endometriotic tissues through mTOR activation.^83^ In endometriosis, the genes and pathways involved in autophagy initiation and regulation (e.g., mTOR, HIF‐1α, C‐X‐C motif chemokine receptor 4 (CXCR4), and estrogen receptor 1 (ESR1)) are upregulated, whereas the downstream ATG‐related genes (e.g., *ULK1* and *BECN1* ^65^, ^66^, ^84^) and microtubule‐associated proteins (e.g., LC3‐I, LC3‐II, and LC3II/LC3I ratio) are often downregulated.^74^, ^85^, ^86^ Indeed, it is often characterized by decreased number of autophagosomes, decreased conversion of LC3‐I to LC3‐II, decreased BECN1 expression, and increased p62 expression compared with the normal endometrium.^5^ Autophagy is downregulated in endometriosis through AKT/mTOR signaling, which inhibits apoptosis^83^ (Figure 1, ①). This suggests that autophagy is a key positive regulator of endometriotic cell apoptosis.

Furthermore, the interlinked connections between autophagy and mitophagy have been reported in endometriosis. A decrease in autophagy activity restores the oxidative imbalance by increasing the expression of nuclear factor (erythroid‐derived) 2‐like (NRF2) and the antioxidant proteins NAD(P)H quinone dehydrogenase 1 (NQO1) and heme oxygenase 1 (HO1).^50^ Inhibiting autophagy also restores mitophagy homeostasis and significantly increases the levels of Parkin in a rat model of endometriosis.^50^ Therefore, well‐coordinated quality control mechanisms are essential for mitochondrial homeostasis and adaptation to stress, such as impaired autophagy and apoptosis.

### 5.2 Hypoxia

When endometrial cells are shed into the peritoneal cavity during menstruation, they face hypoxia. Autophagy is initiated under such stressful conditions and are particularly common in cancer lesions.^87^, ^88^ In patients with endometriosis, autophagic vacuoles and autophagosomes accumulate extensively within ectopic endometrial cells under hypoxic conditions.^3^ Hypoxia leads to stabilization of HIF‐1α, which upregulates autophagy through activation of the downstream gene *BNIP3* ^58^ (Figure 1, ②; Figure 2, ②, ③, and ④). HIF‐1α promotes autophagy and attenuates apoptosis.^3^, ^89^ HIF‐1α also upregulates Bcl‐2 expression.^90^ Beclin1 is downregulated by competing with Bcl‐2 for interaction with Bnip3. HIF‐1α was shown to enhance the migration and invasion of human endometriotic stromal cells through upregulation of autophagy and Bcl‐2 expression^3^, ^89^ (Figure 1, ③), which may contribute to the pathogenesis of endometriosis by reducing the apoptosis of endometriotic cells. Thus, autophagy is thought of as a survival mechanism that can prevent cell death or apoptosis under hypoxia.^3^ Conversely, excessive autophagy can accelerate cell death. Indeed, ectopic endometrial tissues also show increased LC3 levels and decreased p62 levels, which lead to apoptosis^3^ (Figure 1, ④). Therefore, autophagy may switch from a survival to a cell death program or vice versa based on the concentrations of Bcl‐2, Beclin1, and Bnip3 in a hypoxic environment.

### 5.3 Oxidative stress

The accumulation of high levels of hemoglobin, heme, and iron is caused by cyclic bleeding found in patients with endometriosis.^91^ Heme is catabolized to biliverdin, carbon monoxide, and iron by the heme oxygenase enzyme system.^92^ In fact, the concentrations of iron within endometriosis cysts have been reported to vary between 65.3 and 1046.3 mg/L, demonstrating that the degree of iron‐induced oxidative stress also varies depending on endometriotic foci.^90^ Excess iron accumulated in ovarian endometriotic lesions is strongly associated with an oxidative stress‐induced autophagic stimulus^93^ (Figure 1, ⑤). Iron excess is believed to generate high levels of ROS and oxidative stress through the Fenton reaction, which significantly inhibits cell proliferation and causes cell death.^94^ Also, iron overload causes extensive apoptosis and induces cytoplasmic vacuolization as the morphological changes along with increased LC3‐II levels (Figure 1, ④).^95^ Therefore, the overactivation of autophagy and apoptosis under oxidative stress may negatively impact normal endometrial growth and the development of endometriosis.

On the other hand, activation of autophagy may also exert cytoprotective properties in endometriosis.^67^ Indeed, oxygen‐derived‐free radicals formed by excess labile iron are important modulators of Nrf2, nuclear factor kappa‐light‐chain‐enhancer of activated B cells (NF‐κB), AMP‐activated protein kinase (AMPK), Akt, and p53.^94^ For example, activation of the Nrf2 pathway upregulates the expression of cytoprotective genes and antioxidant genes, reduces the level of ROS, protects cells from ROS, and blocks apoptosis^94^, ^96^ (Figure 1, ⑥). Furthermore, autophagy was shown to promote the survival of eutopic endometrial stromal cells by reducing ROS generation possibly through mediating mitochondrial quality control^93^ (Figure 1, ⑥ and ⑦). In addition, iron overload causes activation of protective autophagy and inhibition of apoptosis in eutopic endometrial stromal cells through upregulation of sirtuin 1 (SIRT1) expression^93^ (Figure 1, ⑦ and ③). SIRT1 participates in the regulation of autophagy through deacetylation of specific autophagy‐related proteins (e.g., Beclin1 and LC3).^97^ Altered expression of autophagy‐related molecules (e.g., Bcl‐2, Bax, Beclin1, Bnip3, Parkin, LC3, and p62) in endometriotic cells can switch the signaling from pro‐apoptosis to antiapoptosis (Figure 1, ④ vs ③). Therefore, contradictory findings may exist in endometriosis, as iron‐induced oxidative stress exhibits a dual role in cell‐fate determination, i.e., cell death and cytoprotection, based on the concentration of iron in endometriotic cysts.

### 5.4 Estrogen

Estrogen plays a crucial role in the pathogenesis of endometriosis. Genes involved in the initiation and regulation of autophagy (e.g., mTOR, CXCR4, and ESR1) are upregulated in endometriosis.^73^ Estrogen receptor signaling activates signaling pathways such as PI3K/AKT/mTOR and C‐X‐C motif chemokine ligand 12 (CXCL12)‐CXCR4 axis.^73^ Therefore, estrogen suppresses autophagy through mTOR signal activation and CXCL12/CXCR4 interaction and promotes endometrial stromal cell proliferation^73^ (Figure 1, ①). Mechanistically, similar to mTOR, CXCL12 may inhibit autophagy by reducing Beclin1 expression, reducing LC3B‐I to LC3B‐II conversion, increasing p62 levels, and downregulating autophagosome.^98^ High estrogen levels and progesterone resistance, a hallmark of endometriosis, may be involved in the downregulation of autophagy.^5^ Therefore, estrogen could decrease the apoptosis through the inhibition of autophagy.

### 5.5 AMPK

Endometriotic cells adapt to various environmental stressors and survive under nutrient deprivation and starvation. AMPK is a metabolic sensor that responds to low cellular energy stores, thereby allowing the cell to maintain energy homeostasis.^99^ Activation of AMPK inhibits mTOR via inactivation of the PI3K/Akt signaling pathway^99^, ^100^ (Figure 1, ①). AMPK induces autophagy by inhibiting mechanistic target of rapamycin complex 1 (MTORC1), activating the ULK1 complex, and phosphorylating Beclin1.^101^, ^102^ AMPK‐mediated autophagy is a cytoprotective mechanism that increases nutrient and energy demand.^99^

### 5.6 P53

p53 is thought to inhibit mTOR activity, leading to the induction of autophagic and apoptotic pathways (Figure 1, ① and ②).^11^, ^103^, ^104^ Decreased p53 expression in ovarian endometrioma suggests inhibition of apoptosis.^76^

### 5.7 Others

In addition to these pathways or stressors, many genes that control autophagy and apoptosis in endometriosis have been reported. For example, Mst1 is known as a major growth suppressor involved in cancer invasion, proliferation, and apoptosis.^51^ Mst1 activates mitochondrial fission and inhibits mitophagy through enhancing Drp1 activation and repressing p53‐mediated Parkin transcription activity.^51^ Downregulation of Mst1 expression in endometriosis has been reported to promote endometriotic cell proliferation through activation of mitophagy.^51^ Yes‐associated protein (YAP), a core effector component of the Hippo signaling pathway, is associated with organogenesis, malignancy, and endometriosis.^105^ The Hippo‐YAP pathway promotes cell proliferation in endometriotic stromal cells through inhibition of autophagy and apoptosis.^86^ The cyclic GMP‐AMP synthase (cGAS)‐stimulator of interferon genes (STING) pathway promotes immune effector responses associated with tumorigenesis.^106^ Activation of the cGAS‐STING signaling pathway in endometriosis causes cell proliferation through induction of autophagy.^107^ The tumor suppressor DIRAS family GTPase 3 (DIRAS3) is a physiological autophagy inducer.^108^ Upregulation of DIRAS3 expression in endometriosis promotes proliferation of endometriotic epithelial cells through activating autophagy and inhibiting apoptosis.^109^ The tumor suppressor programmed cell death 4 (PDCD4) suppresses tumor progression.^110^ PDCD4 partially suppresses endometriosis cell proliferation and invasion through inhibition of autophagy.^110^

### 5.8 The relationship between autophagy and apoptosis in animal models of endometriosis

Animal models are essential to better understand the molecular mechanisms involved in autophagy and apoptosis in endometriosis.^15^, ^50^, ^75^, ^77^, ^107^, ^111^, ^112^ In this section, rather than providing a detailed overview of animal models of endometriosis, we explain the contradictory role of autophagy. Most experimental endometriosis was induced by transplantation of normal uterine tissue into the peritoneal cavity.^15^, ^75^ The autophagic pathway was altered in the endometriosis‐like lesions as compared with eutopic endometrium and normal endometrium.^75^ In a rat model of endometriosis, the expression of mTOR was increased, and the expression of Beclin1, Bnip3, Ambra1, LC3II, and Parkin was decreased,^15^ suggesting that the autophagy level is decreased in ectopic endometrium compared to eutopic endometrium and normal endometrium. Conversely, there were also reports that autophagy markers (e.g., Beclin1 and LC3B) were elevated in the ectopic endometrium and further increased in the eutopic endometrium from a mouse model of endometriosis compared to controls.^75^ This model suggests that activation of autophagy in endometriotic cells may favor apoptosis inhibition. Collectively, similar to in vitro and clinical data, the resulting data in animal models of endometriosis are also inconsistent. Interestingly, changes in Beclin1 and Bnip3 expression were also identified in animal models.

## 6 MODULATING AUTOPHAGY AS A THERAPEUTIC STRATEGY FOR ENDOMETRIOSIS

Only one paper was found that discussed pharmacological modulation of mitophagy and the therapeutic potential of targeting mitochondrial dynamics in endometriosis.^51^ Therefore, this section summarizes therapeutic potential of targeting autophagy in the treatment of endometriosis. Accumulating evidence indicated that pharmacological modulation of autophagy attenuated the progression of endometriosis in both in vitro and in vivo settings. However, as shown below, research has shown varied results on the autophagy‐mediated apoptosis properties, including that autophagy‐mediated apoptosis induction inhibited the development and progression of endometriotic lesions,^15^, ^50^, ^74^, ^112^ whereas autophagy inhibition significantly reduced the proliferation of endometriotic cells through promoting apoptosis.^3^, ^67^, ^75^

Several papers have reported that autophagy results in apoptosis induction in endometriotic cells.^15^, ^50^, ^74^, ^112^ First, rapamycin, a macrocyclic antibiotic isolated from *Streptomyces hygroscopicus*, is an immunosuppressant, antifungal, and antitumor drug that blocks mTOR protein kinase.^113^ Induction of autophagy by rapamycin affects apoptosis in a variety of cell types.^33^ Choi et al reported that rapamycin induces autophagy and further promotes apoptosis in endometriotic cyst stromal cells.^74^ Mechanistically, the mTOR inhibition acts as a hub to activate autophagy mechanisms through increased expression of Beclin1, LC3II, Bnip3, Ambra1, and Parkin in a rat model of endometriosis.^15^ Furthermore, rapamycin‐mediated activation of mitophagy induces apoptosis by activating proapoptotic Bcl‐2 family proteins (e.g., Bax), inducing cytochrome c release, and then promoting caspase activation.^30^, ^74^ Thus, upregulated autophagy triggered by rapamycin can promote apoptosis.^74^, ^81^ Although rapamycin can provide a therapeutic treatment for endometriosis, its efficacy may be limited because of toxicity, side effects, and a ubiquitous expression of mTOR.

Second, the search for and development of novel mTOR modulators with fewer side effects to treat endometriosis remains a challenge. In recent years, plant‐based natural products have attracted attention. Açai Berry is an Amazon's popular functional food produced by the Euterpe oleracea palm and has been reported as a molecule that modulates the autophagy pathway.^114^ Açai Berry has demonstrated its efficacy in animal models of endometriosis.^50^ Açai Berry inhibits PI3K/Akt/extracellular‐regulated MAP kinase (ERK)1/2/mTOR signals, promotes the activity of ULK1/Beclin1/Ambra1 molecules, and enhances the processes of the autophagosome nucleation, expansion, and maturation.^50^ Furthermore, it promotes apoptosis through upregulating Bax expression and downregulating Bcl‐2 expression.^50^

Third, increased estrogen receptor expression and decreased progesterone receptor isoform B expression are negative regulators of apoptosis and autophagy in endometriotic cells.^112^ For example, SCM‐198 is the synthetic compound of leonurine, an alkaloid found in Herba leonuri, and has an antiestrogenic property.^112^ SCM‐198 promotes endometriosis cell death via upregulation of apoptosis.^112^ Furthermore, dienogest treatment induces autophagy and promotes apoptosis through inhibition of Akt/ERK1/2/mTOR signaling in endometriotic cells.^83^ Thus, autophagy induction via mTOR inhibition is emerging as a promising therapeutic strategy for endometriosis.

Conversely, autophagy inhibition may also promise to be an effective strategy for treatment of endometriosis. Hydroxychloroquine (HCQ), an autophagy inhibitor, is an alkalinizing lysosomotropic agent that have been used for the treatment of malaria and various autoimmune diseases such as systemic lupus erythematosus and rheumatic and dermatologic diseases.^75^, ^115^ Bafilomycin A1, a chemical inhibitor of lysosomal proton pump vacuolar‐type ATPase (V‐ATPase), prevents the formation of autophagosomes, blocks autophagosome‐lysosome fusion, inhibits autolysosome acidification, and disrupts autophagic flux, leading to autophagy inhibition.^116^ Treatment with hydroxychloroquine^75^ significantly decreased endometriotic cell growth through promoting apoptosis. Additionally, bafilomycin A1 inhibited autophagy in endometriotic stromal cells.^117^ In addition, HIF‐1α promotes the migration and invasion of endometrial stromal cells through upregulation of the expression of autophagy‐related molecules.^3^ Paeonol, 2′‐hydroxy‐4′‐methoxyacetophenone, is a bioactive phenol present in the root bark of the Moutan Cortex^118^ and downregulates the HIF‐1α‐mediated pathway proteins.^67^ HIF‐1α‐mediated autophagy inhibition by Paeonol suppresses both migration and invasion in endometriotic cells.^67^

Collectively, HIF‐1α and mTOR act as a positive and negative regulator of autophagy, respectively, in endometriosis. The inactivation of the PI3K/Akt/mTOR pathway can induce autophagy and subsequently promotes apoptosis. Conversely, the inactivation of the HIF‐1α pathway can promote apoptosis through inhibiting autophagy. These findings support the view that fine‐tuning the induction and inhibition of autophagy can be used as an effective intervention strategy for endometriosis treatment.

## 7 DISCUSSION

We highlighted studies that have evaluated alterations in autophagy/mitophagy and apoptosis in endometriosis and provided an overview regarding its pathogenesis and personalized treatment strategies. Autophagy and mitophagy have been reported to control apoptosis through various pathways and contribute to promoting the development and progression of endometriosis.^5^ Since endometriosis is characterized by iron‐mediated oxidative stress, mitophagy has been studied in the context of regulatory networks that coordinate mitochondrial quality control and antioxidant capacity. However, research on mitophagy in endometriosis is limited, and only a few studies have addressed the ubiquitin‐independent mitophagy pathway. Although there is evidence suggesting that ubiquitin‐independent pathway may be a critical factor in mitophagy, their role in the development, progression, and pathogenesis of endometriosis remains unclear. Therefore, this review mainly focuses on autophagy. Previous studies on the contradictory outcomes of the autophagy‐mediated apoptosis have facilitated an elucidation of the underlying mechanisms regulating autophagy and apoptosis. Endometriotic cells can switch their autophagic responses from inhibitory to promoting mechanism or vice versa to adapt to ever‐changing environments, such as the regulation of signal pathway and oxygen, iron, and nutrient concentrations (Figure 1, ① and ⑤). Upregulation of mTOR expression can suppress autophagy,^15^, ^33^, ^50^, ^61^, ^73^, ^74^, ^100^, ^112^ whereas hypoxia and iron‐mediated oxidative stress often promote autophagy.^3^, ^25^, ^62^ For example, inappropriate activation of the PI3K/Akt/ERK1/2/mTOR pathway leads to inhibition of autophagy and subsequent suppression of apoptosis (Figure 1, ①). Inhibition of autophagy suppresses apoptosis and promotes endometriotic cell proliferation. Indeed, some drugs, such as dienogest,^83^ rapamycin,^15^, ^33^, ^74^ Açai Berry,^50^ and HCQ,^75^ have been reported to exhibit apoptosis promoting and growth suppressive effects on endometriosis by promoting autophagy. On the other hand, previous studies have also shown that ovarian endometriotic cells upregulate autophagy to survive and promote growth.^76^, ^119^ In fact, activation of the HIF‐1α pathway and excess ROS induces autophagy and avoids cell death through antiapoptotic effects (Figure 1, ②, ⑤, ⑦, and ③). The autophagy pathways that regulate cell homeostasis are crucial for scavenging damaged organelles, lipids, proteins, and DNA.^26^, ^27^, ^28^ Therefore, endometriotic cells must rely on autophagy to survive external challenges such as hypoxia and oxidative stress. However, HIF‐1α‐ or ROS‐induced overactivation of autophagy has also been reported to induce cell death via promoting apoptosis (Figure 1, ④). Therefore, autophagy may play a dual role in suppressing and promoting the development of endometriosis.

We discuss why these seemingly contradictory results occur. First, regarding the mechanism by which autophagy controls apoptosis, alternative or compensatory mechanisms need to be considered. For example, oxidative stress and nutrient starvation induce the expression of SIRT1^120^ and AMPK^121^ as compensatory mechanisms as a means of coping with harsh environments. These molecules have key roles in the regulation of cellular metabolism and homeostasis. Key signaling pathways and downstream target molecules involved in autophagy are significantly altered in each endometriosis to deal with ever‐changing environments.^5^ Second, autophagy and apoptosis are tightly regulated processes that share a common signal.^6^, ^15^, ^29^, ^122^ Bcl‐2, Bax, Beclin1, and Bnip3 are important molecules that regulate two distinct cellular processes, autophagy and apoptosis.^35^, ^36^, ^37^, ^55^, ^61^, ^62^, ^63^, ^64^ Beclin1 and Bnip3 can bind Bcl‐2 in a mutually exclusive manner and are involved in determining autophagy and apoptosis (see Section 4). Therefore, apoptosis may be promoted or inhibited depending on the concentrations of Bcl‐2, Bax, Beclin1, and Bnip3 proteins in endometriotic lesions (Figure 2). These divergent outcomes are likely due to the unique combination of the altered expression of multiple downstream molecules and associated pathways in each endometriotic cell.

Finally, studies using endometriosis samples are important for the development of new therapeutic strategies for targeting autophagy. At present, when and how cells choose cytoprotection or apoptosis remains unclear. In some lesions, endometriotic cells can be eliminated by autophagy, so autophagy‐promoting drugs may be helpful in the treatment (Figure 1, ①). In other lesions, antiautophagic drugs may result in therapeutic effects against endometriosis possibly through inhibiting the autophagy‐mediated quality control and restoring apoptosis (Figure 1, ④). In particular, strategies that modulate the expression level of autophagy (i.e., the transition from cytoprotection to apoptosis) could be a potential target for endometriosis. Therefore, there is an urgent need to develop novel therapies targeting autophagy that respond to changing conditions in real time.

In conclusion, this review summarizes the current understanding of the molecular mechanisms involved in autophagy and apoptosis in the pathogenesis of endometriosis and also discusses the challenges and future therapeutic directions targeting autophagy in endometriosis.

## 8 FUTURE PERSPECTIVES

Endometriotic cells can fine‐tune autophagy pathway to survive in their ever‐changing environments. Currently, it is difficult to monitor the expression of autophagy‐related genes or proteins in individual endometriotic cells in real time. Genomic profiles of circulating cell‐free DNA or exosomes based on liquid biopsy may provide repeated transcriptomic snapshots of lesion heterogeneity. A custom panel needs to be generated for several genes related to autophagy and apoptosis (e.g., *mTOR*, *HIF1A*, *BCL2*, *BECN1*, *BNIP3*, *ULK1*, *LC3I*, *LC3II*, *p62*, *PINK1*, *PRKN*, *AMPK*, *SIRT1*, and *p53*) to allow diagnosis in a single acquisition. For example, altered expression of genes such as *BCL2*, *BECN1*, and *BNIP3* may help identify endometriosis patients who would benefit from antiautophagic or proapoptotic drugs. Furthermore, the degree of overexpression of *mTOR* or *HIF1A* may provide different therapeutic options on autophagy regulation. Such panel‐based genetic diagnosis may improve clinical management of endometriosis patients. Therefore, research focusing on the regulation of autophagy will emerge as a promising therapeutic strategy for endometriosis.

## FUNDING INFORMATION

No funding was received.

## CONFLICT OF INTEREST STATEMENT

The authors declare no competing interests.

## ETHICS STATEMENT

Ethics Approval The submitted paper is a review article and has not been approved by the Institutional Review Board and the Research and Ethical Committee of Nara Medical University Graduate School of Medicine, Kashihara, Japan.

## CONSENT TO PARTICIPATE

Not applicable.

## CONSENT FOR PUBLICATION

Not applicable.

## Data Availability

No new data were created.

## References

[1] Giudice LC . Clinical practice. Endometriosis. N Engl J Med. 2010;362(25):2389–2398. 10.1056/NEJMcp1000274 20573927 PMC3108065

[2] Sampson JA . Peritoneal endometriosis due to the menstrual dissemination of endometrial tissue into the peritoneal cavity. Am J Obstet Gynecol. 1927;14:422–469.

[3] Liu H , Zhang Z , Xiong W , Zhang L , Du Y , Liu Y , et al. Long non‐coding RNA MALAT1 mediates hypoxia‐induced pro‐survival autophagy of endometrial stromal cells in endometriosis. J Cell Mol Med. 2019;23(1):439–452. 10.1111/jcmm.13947 30324652 PMC6307811

[4] Taniguchi F , Kaponis A , Izawa M , Kiyama T , Deura I , Ito M , et al. Apoptosis and endometriosis. Front Biosci. 2011;3:648–662. 10.2741/e277 21196342

[5] Yang HL , Mei J , Chang KK , Zhou WJ , Huang LQ , Li MQ . Autophagy in endometriosis. Am J Transl Res. 2017;9(11):4707–4725. 29218074 PMC5714760

[6] Maiuri MC , Zalckvar E , Kimchi A , Kroemer G . Self‐eating and self‐killing: crosstalk between autophagy and apoptosis. Nat Rev Mol Cell Biol. 2007;8(9):741–752. 10.1038/nrm2239 17717517

[7] Shen HH , Zhang T , Yang HL , Lai ZZ , Zhou WJ , Mei J , et al. Ovarian hormones‐autophagy‐immunity axis in menstruation and endometriosis. Theranostics. 2021;11(7):3512–3526. 10.7150/thno.55241 33537101 PMC7847674

[8] Klionsky DJ , Emr SD . Autophagy as a regulated pathway of cellular degradation. Science. 2000;290:1717–1721. 10.1126/science.290.5497.1717 11099404 PMC2732363

[9] He C , Klionsky DJ . Regulation mechanisms and signaling pathways of autophagy. Annu Rev Genet. 2009;43:67–93. 10.1146/annurev-genet-102808-114910 19653858 PMC2831538

[10] Su Y , Zhang JJ , He JL , Liu XQ , Chen XM , Ding YB , et al. Endometrial autophagy is essential for embryo implantation during early pregnancy. J Mol Med (Berl). 2020;98(4):555–567. 10.1007/s00109-019-01849-y 32072231

[11] Gupta R , Ambasta RK , Kumar P . Autophagy and apoptosis cascade: which is more prominent in neuronal death? Cell Mol Life Sci. 2021;78(24):8001–8047. 10.1007/s00018-021-04004-4 34741624 PMC11072037

[12] Ventruti A , Cuervo AM . Autophagy and neurodegeneration. Curr Neurol Neurosci Rep. 2007;7:443–451. 10.1007/s11910-007-0068-5 17764636

[13] Levine B , Kroemer G . Autophagy in the pathogenesis of disease. Cell. 2008;132(1):27–42. 10.1016/j.cell.2007.12.018 18191218 PMC2696814

[14] Bednarczyk M , Zmarzły N , Grabarek B , Mazurek U , Muc‐Wierzgoń M . Genes involved in the regulation of different types of autophagy and their participation in cancer pathogenesis. Oncotarget. 2018;9:34413–34428. 10.18632/oncotarget.26126 30344951 PMC6188136

[15] Siracusa R , D'Amico R , Impellizzeri D , Cordaro M , Peritore AF , Gugliandolo E , et al. Autophagy and mitophagy promotion in a rat model of endometriosis. Int J Mol Sci. 2021;22(10):5074. 10.3390/ijms22105074 34064854 PMC8150724

[16] Green DR , Galluzzi L , Kroemer G . Mitochondria and the autophagy‐inflammation‐cell death axis in organismal aging. Science. 2011;333(6046):1109–1112. 10.1126/science.1201940 21868666 PMC3405151

[17] Kobayashi H , Matsubara S , Yoshimoto C , Shigetomi H , Imanaka S . The role of mitochondrial dynamics in the pathophysiology of endometriosis. J Obstet Gynaecol Res. 2023;49(12):2783–2791. 10.1111/jog.15791 37681703

[18] Su L , Zhang J , Gomez H , Kellum JA , Peng Z . Mitochondria ROS and mitophagy in acute kidney injury. Autophagy. 2023;19(2):401–414. 10.1080/15548627.2022.2084862 35678504 PMC9851232

[19] Ashrafi G , Schwarz TL . The pathways of mitophagy for quality control and clearance of mitochondria. Cell Death Differ. 2013;20(1):31–42. 10.1038/cdd.2012.81 22743996 PMC3524633

[20] Gurung P , Lukens JR , Kanneganti TD . Mitochondria: diversity in the regulation of the NLRP3 inflammasome. Trends Mol Med. 2015;21(3):193–201. 10.1016/j.molmed.2014.11.008 25500014 PMC4352396

[21] Wang K . Autophagy and apoptosis in liver injury. Cell Cycle. 2015;14(11):1631–1642. 10.1080/15384101.2015.1038685 25927598 PMC4614283

[22] Zhan L , Li J , Wei B . Autophagy in endometriosis: friend or foe? Biochem Biophys Res Commun. 2018;495(1):60–63. 10.1016/j.bbrc.2017.10.145 29107692

[23] Xie Q , Liu Y , Li X . The interaction mechanism between autophagy and apoptosis in colon cancer. Transl Oncol. 2020;13(12):100871. 10.1016/j.tranon.2020.100871 32950931 PMC7509232

[24] White E . Autophagy and p53. Cold Spring Harb Perspect Med. 2016;6(4):a026120. 10.1101/cshperspect.a026120 27037419 PMC4817743

[25] Hu YL , DeLay M , Jahangiri A , Molinaro AM , Rose SD , Carbonell WS , et al. Hypoxia‐induced autophagy promotes tumor cell survival and adaptation to antiangiogenic treatment in glioblastoma. Cancer Res. 2012;72(7):1773–1783. 10.1158/0008-5472.CAN-11-3831 22447568 PMC3319869

[26] Wang Y , Nartiss Y , Steipe B , McQuibban GA , Kim PK . ROS‐induced mitochondrial depolarization initiates PARK2/PARKIN‐dependent mitochondrial degradation by autophagy. Autophagy. 2012;8(10):1462–1476. 10.4161/auto.21211 22889933

[27] Glick D , Barth S , Macleod KF . Autophagy: cellular and molecular mechanisms. J Pathol. 2010;221(1):3–12. 10.1002/path.2697 20225336 PMC2990190

[28] Yang YP , Liang ZQ , Gu ZL , Qin ZH . Molecular mechanism and regulation of autophagy. Acta Pharmacol Sin. 2005;26(12):1421–1434. 10.1111/j.1745-7254.2005.00235.x 16297339

[29] Galluzzi L , Vicencio JM , Kepp O , Tasdemir E , Maiuri MC , Kroemer G . To die or not to die: that is the autophagic question. Curr Mol Med. 2008;8(2):78–91. 10.2174/156652408783769616 18336289

[30] Wanderoy S , Hees JT , Klesse R , Edlich F , Harbauer AB . Kill one or kill the many: interplay between mitophagy and apoptosis. Biol Chem. 2020;402(1):73–88. 10.1515/hsz-2020-0231 33544491

[31] Feng Y , He D , Yao Z , Klionsky DJ . The machinery of macroautophagy. Cell Res. 2014;24(1):24–41. 10.1038/cr.2013.168 24366339 PMC3879710

[32] Dunlop EA , Tee AR . mTOR and autophagy: a dynamic relationship governed by nutrients and energy. Semin Cell Dev Biol. 2014;36:121–129. 10.1016/j.semcdb.2014.08.006 25158238

[33] Schmelzle T , Hall MN . TOR, a central controller of cell growth. Cell. 2000;103(2):253–262. 10.1016/s0092-8674(00)00117-3 11057898

[34] Jung CH , Jun CB , Ro SH , Kim YM , Otto NM , Cao J , et al. ULK‐Atg13‐FIP200 complexes mediate mTOR signaling to the autophagy machinery. Mol Biol Cell. 2009;20(7):1992–2003. 10.1091/mbc.e08-12-1249 19225151 PMC2663920

[35] He C , Levine B . The Beclin 1 interactome. Curr Opin Cell Biol. 2010;22(2):140–149. 10.1016/j.ceb.2010.01.001 20097051 PMC2854269

[36] Kang R , Zeh HJ , Lotze MT , Tang D . The Beclin 1 network regulates autophagy and apoptosis. Cell Death Differ. 2011;18(4):571–580. 10.1038/cdd.2010.191 21311563 PMC3131912

[37] Zhang J , Ney PA . Role of BNIP3 and NIX in cell death, autophagy, and mitophagy. Cell Death Differ. 2009;16(7):939–946. 10.1038/cdd.2009.16 19229244 PMC2768230

[38] Levine B , Mizushima N , Virgin HW . Autophagy in immunity and inflammation. Nature. 2011;469(7330):323–335. 10.1038/nature09782 21248839 PMC3131688

[39] Kabeya Y , Mizushima N , Ueno T , Yamamoto A , Kirisako T , Noda T , et al. LC3, a mammalian homologue of yeast Apg8p, is localized in autophagosome membranes after processing. EMBO J. 2000;19(21):5720–5728. 10.1093/emboj/19.21.5720 11060023 PMC305793

[40] Youle RJ , van der Bliek AM . Mitochondrial fission, fusion, and stress. Science. 2012;337(6098):1062–1065. 10.1126/science.1219855 22936770 PMC4762028

[41] Adebayo M , Singh S , Singh AP , Dasgupta S . Mitochondrial fusion and fission: the fine‐tune balance for cellular homeostasis. FASEB J. 2021;35(6):e21620. 10.1096/fj.202100067R 34048084 PMC8415099

[42] Schrepfer E , Scorrano L . Mitofusins, from mitochondria to metabolism. Mol Cell. 2016;61(5):683–694. 10.1016/j.molcel.2016.02.022 26942673

[43] Otera H , Ishihara N , Mihara K . New insights into the function and regulation of mitochondrial fission. Biochim Biophys Acta. 2013;1833(5):1256–1268. 10.1016/j.bbamcr.2013.02.002 23434681

[44] Burté F , Carelli V , Chinnery PF , Yu‐Wai‐Man P . Disturbed mitochondrial dynamics and neurodegenerative disorders. Nat Rev Neurol. 2015;11(1):11–24. 10.1038/nrneurol.2014.228 25486875

[45] Nunnari J , Suomalainen A . Mitochondria: in sickness and in health. Cell. 2012;148(6):1145–1159. 10.1016/j.cell.2012.02.035 22424226 PMC5381524

[46] Vyas S , Zaganjor E , Haigis MC . Mitochondria and cancer. Cell. 2016;166(3):555–566. 10.1016/j.cell.2016.07.002 27471965 PMC5036969

[47] Kataoka T . Biological properties of the BCL‐2 family protein BCL‐RAMBO, which regulates apoptosis, mitochondrial fragmentation, and mitophagy. Front Cell Dev Biol. 2022;10:1065702. 10.3389/fcell.2022.1065702 36589739 PMC9800997

[48] Deng Y , Lou T , Kong L , Liu C . Prohibitin2/PHB2, transcriptionally regulated by GABPA, inhibits cell growth via PRKN/Parkin‐dependent mitophagy in endometriosis. Reprod Sci. 2023;30(12):3629–3640. 10.1007/s43032-023-01316-7 37587393

[49] Ji X , Huang C , Mao H , Zhang Z , Zhang X , Yue B , et al. Identification of immune‐ and autophagy‐related genes and effective diagnostic biomarkers in endometriosis: a bioinformatics analysis. Ann Transl Med. 2022;10(24):1397. 10.21037/atm-22-5979 36660690 PMC9843312

[50] D'Amico R , Impellizzeri D , Cordaro M , Siracusa R , Interdonato L , Marino Y , et al. Complex interplay between autophagy and oxidative stress in the development of endometriosis. Antioxidants (Basel). 2022;11(12):2484. 10.3390/antiox11122484 36552692 PMC9774576

[51] Zhao Q , Ye M , Yang W , Wang M , Li M , Gu C , et al. Effect of Mst1 on endometriosis apoptosis and migration: role of Drp1‐related mitochondrial fission and Parkin‐required mitophagy. Cell Physiol Biochem. 2018;45(3):1172–1190. 10.1159/000487450 29448246

[52] McWilliams TG , Muqit MM . PINK1 and Parkin: emerging themes in mitochondrial homeostasis. Curr Opin Cell Biol. 2017;45:83–91. 10.1016/j.ceb.2017.03.013 28437683

[53] Jin SM , Lazarou M , Wang C , Kane LA , Narendra DP , Youle RJ . Mitochondrial membrane potential regulates PINK1 import and proteolytic destabilization by PARL. J Cell Biol. 2010;191(5):933–942. 10.1083/jcb.201008084 21115803 PMC2995166

[54] Zhang T , Xue L , Li L , Tang C , Wan Z , Wang R , et al. BNIP3 protein suppresses PINK1 kinase proteolytic cleavage to promote mitophagy. J Biol Chem. 2016;291(41):21616–21629. 10.1074/jbc.M116.733410 27528605 PMC5076832

[55] Zhou X , Zhao X , Zhou W , Qi H , Zhang H , Ting‐Li Han TL , et al. Impaired placental mitophagy and oxidative stress are associated with dysregulated BNIP3 in preeclampsia. Sci Rep. 2021;11(1):20469. 10.1038/s41598-021-99 837-1 34650122 PMC8516954

[56] Narendra D , Tanaka A , Suen DF , Youle RJ . Parkin is recruited selectively to impaired mitochondria and promotes their autophagy. J Cell Biol. 2008;183(5):795–803. 10.1083/jcb.200809125 19029340 PMC2592826

[57] Lee Y , Lee HY , Hanna RA , Gustafsson ÅB . Mitochondrial autophagy by Bnip3 involves Drp1‐mediated mitochondrial fission and recruitment of Parkin in cardiac myocytes. Am J Physiol Heart Circ Physiol. 2011;301(5):H1924–H1931. 10.1152/ajpheart.00368.2011 21890690 PMC3213962

[58] Xie Y , Liu J , Kang R , Tang D . Mitophagy receptors in tumor biology. Front Cell Dev Biol. 2020;8:594203. 10.3389/fcell.2020.594203 33262988 PMC7686508

[59] Terešak P , Lapao A , Subic N , Boya P , Elazar Z , Simonsen A . Regulation of PRKN‐independent mitophagy. Autophagy. 2022;18(1):24–39. 10.1080/15548627.2021.1888244 33570005 PMC8865282

[60] Liu L , Sakakibara K , Chen Q , Okamoto K . Receptor‐mediated mitophagy in yeast and mammalian systems. Cell Res. 2014;24(7):787–795. 10.1038/cr.2014.75 24903109 PMC4085769

[61] Mazure NM , Pouyssegur J . Atypical BH3‐domains of BNIP3 and BNIP3L lead to autophagy in hypoxia. Autophagy. 2009;5(6):868–869. 10.4161/auto.9042 19587545

[62] Bellot G , Garcia‐Medina R , Gounon P , Chiche J , Roux D , Pouysségur J , et al. Hypoxia‐induced autophagy is mediated through hypoxia‐inducible factor induction of BNIP3 and BNIP3L via their BH3 domains. Mol Cell Biol. 2009;29(10):2570–2581. 10.1128/MCB.00166-09 19273585 PMC2682037

[63] Hollville E , Carroll RG , Cullen SP , Martin SJ . Bcl‐2 family proteins participate in mitochondrial quality control by regulating Parkin/PINK1‐dependent mitophagy. Mol Cell. 2014;55(3):451–466. 10.1016/j.molcel.2014.06.001 24999239

[64] Zhu Y , Massen S , Terenzio M , Lang V , Chen‐Lindner S , Eils R , et al. Modulation of serines 17 and 24 in the LC3‐interacting region of Bnip3 determines pro‐survival mitophagy versus apoptosis. J Biol Chem. 2013;288(2):1099–1113. 10.1074/jbc.M112.399345 23209295 PMC3542995

[65] Kong Z , Yao T . Role for autophagy‐related markers Beclin‐1 and LC3 in endometriosis. BMC Womens Health. 2022;22(1):264. 10.1186/s12905-022-01850-7 35768796 PMC9245300

[66] Zhang L , Liu Y , Xu Y , Wu H , Wei Z , Cao Y . The expression of the autophagy gene beclin‐1 mRNA and protein in ectopic and eutopic endometrium of patients with endometriosis. Int. J Fertil Steril. 2015;8(4):429–436. 10.22074/ijfs.2015.4183 PMC4355304 25780525

[67] Pang C , Wu Z , Xu X , Yang W , Wang X , Qi Y . Paeonol alleviates migration and invasion of endometrial stromal cells by reducing HIF‐1α‐regulated autophagy in endometriosis. Front Biosci (Landmark ed). 2021;26(9):485–495. 10.52586/4961 34590461

[68] Kirmizi DA , Baser E , Okan A , Doganyigit Z . Receptivity, autophagy, and implantation in endometriosis; does antioxidant work? An experimental study. J Food Biochem. 2022;46(10):e14276. 10.1111/jfbc.14276 35712902

[69] Ding Y , Zhu Q , He Y , Lu Y , Wang Y , Qi J , et al. Induction of autophagy by Beclin‐1 in granulosa cells contributes to follicular progesterone elevation in ovarian endometriosis. Transl Res. 2021;227:15–29. 10.1016/j.trsl.2020.06.013 32640290

[70] Kim SI , Yeo SG , Gen Y , Ju HR , Kim SH , Park DC . Differences in autophagy‐associated mRNAs in peritoneal fluid of patients with endometriosis and gynecologic cancers. Eur J Obstet Gynecol Reprod Biol X. 2019;2:100016. 10.1016/j.eurox.2019.100016 31396598 PMC6683980

[71] Zheng J , Luo X , Bao J , Huang X , Jin Y , Chen L , et al. Decreased expression of HOXA10 may activate the autophagic process in ovarian endometriosis. Reprod Sci. 2018;25(9):1446–1454. 10.1177/1933719118768704 29658437

[72] Choi J , Jo M , Lee E , Oh YK , Choi D . The role of autophagy in human endometrium. Biol Reprod. 2012;86(3):70. 10.1095/biolreprod.111.096206 22088918

[73] Mei J , Zhu XY , Jin LP , Duan ZL , Li DJ , Li MQ . Estrogen promotes the survival of human secretory phase endometrial stromal cells via CXCL12/CXCR4 up‐regulation‐mediated autophagy inhibition. Hum Reprod. 2015;30(7):1677–1689. 10.1093/humrep/dev100 25976655

[74] Choi J , Jo M , Lee E , Kim HJ , Choi D . Differential induction of autophagy by mTOR is associated with abnormal apoptosis in ovarian endometriotic cysts. Mol Hum Reprod. 2014;20(4):309–317. 10.1093/molehr/gat091 24319109

[75] Ruiz A , Rockfield S , Taran N , Haller E , Engelman RW , Flores I , et al. Effect of hydroxychloroquine and characterization of autophagy in a mouse model of endometriosis. Cell Death Dis. 2016;7(1):e2059. 10.1038/cddis.2015.361 26775710 PMC4816166

[76] Allavena G , Carrarelli P , Del Bello B , Luisi S , Petraglia F , Maellaro E . Autophagy is upregulated in ovarian endometriosis: a possible interplay with p53 and heme oxygenase‐1. Fertil Steril. 2015;103(5):1244–1251.e1. 10.1016/j.fertnstert.2015.02.007 25772769

[77] Li H , Yang H , Lu S , Wang X , Shi X , Mao P . Autophagy‐dependent ferroptosis is involved in the development of endometriosis. Gynecol Endocrinol. 2023;39(1):2242962. 10.1080/09513590.2023.2242962 37553011

[78] Honda H , Barrueto FF , Gogusev J , Im DD , Morin PJ . Serial analysis of gene expression reveals differential expression between endometriosis and normal endometrium. Possible roles for AXL and SHC1 in the pathogenesis of endometriosis. Reprod Biol Endocrinol. 2008;6:59. 10.1186/1477-7827-6-59 19055724 PMC2615013

[79] Laudanski P , Szamatowicz J , Kowalczuk O , Kuzmicki M , Grabowicz M , Chyczewski L . Expression of selected tumor suppressor and oncogenes in endometrium of women with endometriosis. Hum Reprod. 2009;24(8):1880–1890. 10.1093/humrep/dep175 19429661

[80] Kim TH , Yu Y , Luo L , Lydon JP , Jeong JW , Kim JJ . Activated AKT pathway promotes establishment of endometriosis. Endocrinology. 2014;155(5):1921–1930. 10.1210/en.2013-1951 24605828 PMC3990849

[81] Leconte M , Nicco C , Ngô C , Chéreau C , Chouzenoux S , Marut W , et al. The mTOR/AKT inhibitor temsirolimus prevents deep infiltrating endometriosis in mice. Am J Pathol. 2011;179(2):880–889. 10.1016/j.ajpath.2011.04.020 21718677 PMC3157265

[82] Yagyu T , Tsuji Y , Haruta S , Kitanaka T , Yamada Y , Kawaguchi R , et al. Activation of mammalian target of rapamycin in postmenopausal ovarian endometriosis. Int J Gynecol Cancer. 2006;16(4):1545–1551. 10.1111/j.1525-1438.2006.00625.x 16884363

[83] Choi J , Jo M , Lee E , Lee DY , Choi D . Dienogest enhances autophagy induction in endometriotic cells by impairing activation of AKT, ERK1/2, and mTOR. Fertil Steril. 2015;104(3):655–664.e1. 10.1016/j.fertnstert.2015.05.020 26051103

[84] Ren Y , Mu L , Ding X , Zheng W . Decreased expression of Beclin 1 in eutopic endometrium of women with adenomyosis. Arch Gynecol Obstet. 2010;282(4):401–406. 10.1007/s00404-009-1280-0 19921231

[85] Sui X , Li Y , Sun Y , Li C , Li X , Zhang G . Expression and significance of autophagy genes LC3, Beclin1 and MMP‐2 in endometriosis. Exp Ther Med. 2018;16(3):1958–1962. 10.3892/etm.2018.6362 30186424 PMC6122207

[86] Pei T , Huang X , Long Y , Duan C , Liu T , Li Y , et al. Increased expression of YAP is associated with decreased cell autophagy in the eutopic endometrial stromal cells of endometriosis. Mol Cell Endocrinol. 2019;491:110432. 10.1016/j.mce.2019.04.012 31014943

[87] Martinez‐Outschoorn UE , Trimmer C , Lin Z , Whitaker‐Menezes D , Chiavarina B , Zhou J , et al. Autophagy in cancer associated fibroblasts promotes tumor cell survival: role of hypoxia, HIF1 induction and NFkappaB activation in the tumor stromal microenvironment. Cell Cycle. 2010;9(17):3515–3533. 10.4161/cc.9.17.12928 20855962 PMC3047617

[88] Wu J , Lei Z , Yu J . Hypoxia induces autophagy in human vascular endothelial cells in a hypoxia‐inducible factor 1‐dependent manner. Mol Med Rep. 2015;11(4):2677–2682. 10.3892/mmr.2014.3093 25514934

[89] Xu TX , Zhao SZ , Dong M , Yu XR . Hypoxia responsive miR‐210 promotes cell survival and autophagy of endometriotic cells in hypoxia. Eur Rev Med Pharmacol Sci. 2016;20(3):399–406. 26914112

[90] Tsuzuki T , Okada H , Shindoh H , Shimoi K , Nishigaki A , Kanzaki H . Effects of the hypoxia‐inducible factor‐1 inhibitor echinomycin on vascular endothelial growth factor production and apoptosis in human ectopic endometriotic stromal cells. Gynecol Endocrinol. 2016;32(4):323–328. 10.3109/09513590.2015.1121225 26654708

[91] Yoshimoto C , Iwabuchi T , Shigetomi H , Kobayashi H . Cyst fluid iron‐related compounds as useful markers to distinguish malignant transformation from benign endometriotic cysts. Cancer Biomark. 2015;15(4):493–499. 10.3233/CBM-150484 25835178 PMC12965089

[92] Iwabuchi T , Yoshimoto C , Shigetomi H , Kobayashi H . Cyst fluid hemoglobin species in endometriosis and its malignant transformation: the role of metallobiology. Oncol Lett. 2016;11(5):3384–3388. 10.3892/ol.2016.4383 27123121 PMC4841012

[93] Zhou Y , Zhao X , Zhang L , Xia Q , Peng Y , Zhang H , et al. Iron overload inhibits cell proliferation and promotes autophagy via PARP1/SIRT1 signaling in endometriosis and adenomyosis. Toxicology. 2022;465:153050. 10.1016/j.tox.2021.153050 34826546

[94] Kaminskyy VO , Zhivotovsky B . Free radicals in cross talk between autophagy and apoptosis. Antioxid Redox Signal. 2014;21(1):86–102. 10.1089/ars.2013.5746 24359220

[95] Bauckman KA , Haller E , Flores I , Nanjundan M . Iron modulates cell survival in a Ras‐ and MAPK‐dependent manner in ovarian cells. Cell Death Dis. 2013;4(4):e592. 10.1038/cddis.2013.87 23598404 PMC3668627

[96] Sadrkhanloo M , Entezari M , Orouei S , Zabolian A , Mirzaie A , Maghsoudloo A , et al. Targeting Nrf2 in ischemia–reperfusion alleviation: from signaling networks to therapeutic targeting. Life Sci. 2022;300:120561. 10.1016/j.lfs.2022.120561 35460707

[97] Deng Z , Sun M , Wu J , Fang H , Cai S , An S , et al. SIRT1 attenuates sepsis‐induced acute kidney injury via Beclin1 deacetylation‐mediated autophagy activation. Cell Death Dis. 2021;12:217. 10.1038/s41419-021-03508-y 33637691 PMC7910451

[98] Zhang Q , Song J , Cao L , Sun M , Xu T , Yang S , et al. RNF113A targeted by miR‐197 promotes proliferation and inhibits autophagy via CXCR4/CXCL12/AKT/ERK/Beclin1 axis in cervical cancer. Exp Cell Res. 2023;428(1):113632. 10.1016/j.yexcr.2023.113632 37164050

[99] Lin SC , Hardie DG . AMPK: sensing glucose as well as cellular energy status. Cell Metab. 2018;27(2):299–313. 10.1016/j.cmet.2017.10.009 29153408

[100] Assaf L , Eid AA , Nassif J . Role of AMPK/mTOR, mitochondria, and ROS in the pathogenesis of endometriosis. Life Sci. 2022;206:120805. 10.1016/j.lfs.2022.120805 35850246

[101] Egan DF , Shackelford DB , Mihaylova MM , Gelino S , Kohnz RA , Mair W , et al. Phosphorylation of ULK1 (hATG1) by AMP‐activated protein kinase connects energy sensing to mitophagy. Science. 2011;331(6016):456–461. 10.1126/science.1196371 21205641 PMC3030664

[102] Kim J , Kim YC , Fang C , Russell RC , Kim JH , Fan W , et al. Differential regulation of distinct Vps34 complexes by AMPK in nutrient stress and autophagy. Cell. 2013;152(1–2):290–303. 10.1016/j.cell.2012.12.016 23332761 PMC3587159

[103] Feng Z , Zhang H , Levine AJ , Jin S . The coordinate regulation of the p53 and mTOR pathways in cells. Proc Natl Acad Sci USA. 2005;102(23):8204–8209. 10.1073/pnas.0502857102 15928081 PMC1142118

[104] Vousden KH , Prives C . Blinded by the light: the growing complexity of p53. Cell. 2009;137(3):413–431. 10.1016/j.cell.2009.04.037 19410540

[105] Pei T , Luo B , Huang W , Liu D , Li Y , Xiao L , et al. Increased expression of YAP inhibited the autophagy level by upregulating mTOR signal in the Eutopic ESCs of endometriosis. Front Endocrinol (Lausanne). 2022;13:813165. 10.3389/fendo.2022.813165 35173685 PMC8842667

[106] Decout A , Katz JD , Venkatraman S , Ablasser A . The cGAS‐STING pathway as a therapeutic target in inflammatory diseases. Nat Rev Immunol. 2021;21(9):548–569. 10.1038/s41577-021-00524-z 33833439 PMC8029610

[107] Zhu S , Chen Q , Sun J , Du W , Chen Z , Yu M , et al. The cGAS‐STING pathway promotes endometriosis by up‐regulating autophagy. Int Immunopharmacol. 2023;117:109644. 10.1016/j.intimp.2022.109644 36878046

[108] Ornelas A , McCullough CR , Lu Z , Zacharias NM , Kelderhouse LE , Gray J , et al. Induction of autophagy by ARHI (DIRAS3) alters fundamental metabolic pathways in ovarian cancer models. BMC Cancer. 2016;16(1):824. 10.1186/s12885-016-2850-8 27784287 PMC5080741

[109] Yotova I , Proestling K , Haslinger I , Witzmann‐Stern M , Widmar B , Kuessel L , et al. DIRAS3 regulates autophagy in an endometriosis epithelial cell line. Reprod Biomed Online. 2023;47(4):103251. 10.1016/j.rbmo.2023.06.006 37598541

[110] Li Y , Wang X , Wang X , Wan L , Liu Y , Shi Y , et al. PDCD4 suppresses proliferation, migration, and invasion of endometrial cells by inhibiting autophagy and NF‐κB/MMP2/MMP9 signal pathway. Biol Reprod. 2018;99(2):360–372. 10.1093/biolre/ioy052 29912279

[111] Jamali N , Zal F , Mostafavi‐Pour Z , Samare‐Najaf M , Poordast T , Dehghanian A . Ameliorative effects of quercetin and metformin and their combination against experimental endometriosis in rats. Reprod Sci. 2021;28(3):683–692. 10.1007/s43032-020-00377-2 33141412

[112] Lin YK , Li YY , Li Y , Li DJ , Wang XL , Wang L , et al. SCM‐198 prevents endometriosis by reversing low autophagy of endometrial stromal cell via balancing ERα and PR signals. Front Endocrinol (Lausanne). 2022;13:858176. 10.3389/fendo.2022.858176 35784569 PMC9245568

[113] Lin X , Han L , Weng J , Wang K , Chen T . Rapamycin inhibits proliferation and induces autophagy in human neuroblastoma cells. Biosci Rep. 2018;38(6):BSR20181822. 10.1042/BSR20181822 30393233 PMC6265625

[114] Impellizzeri D , D'Amico R , Fusco R , Genovese T , Peritore AF , Gugliandolo E , et al. Açai berry mitigates vascular dementia‐induced neuropathological alterations modulating Nrf‐2/Beclin1 pathways. Cells. 2022;11(16):2616. 10.3390/cells11162616 36010690 PMC9406985

[115] Lee SJ , Silverman E , Bargman JM . The role of antimalarial agents in the treatment of SLE and lupus nephritis. Nat Rev Nephrol. 2011;7(12):718–729. 10.1038/nrneph.2011.150 22009248

[116] Mauvezin C , Nagy P , Juhász G , Neufeld TP . Autophagosome‐lysosome fusion is independent of V‐ATPase‐mediated acidification. Nat Commun. 2015;6:7007. 10.1038/ncomms8007 25959678 PMC4428688

[117] Matsuzaki S , Pouly JL , Canis M . In vitro and in vivo effects of MK2206 and chloroquine combination therapy on endometriosis: autophagy may be required for regrowth of endometriosis. Br J Pharmacol. 2018;175(10):1637–1653. 10.1111/bph.14170 29457968 PMC5913408

[118] Choy KW , Murugan D , Mustafa MR . Natural products targeting ER stress pathway for the treatment of cardiovascular diseases. Pharmacol Res. 2018;132:119–129. 10.1016/j.phrs.2018.04.013 29684674

[119] Liu H , Zhang Z , Xiong W , Zhang L , Xiong Y , Li N , et al. Hypoxia‐inducible factor‐1α promotes endometrial stromal cells migration and invasion by upregulating autophagy in endometriosis. Reproduction. 2017;153(6):809–820. 10.1530/REP-16-0643 28348069 PMC5489654

[120] Santos L , Escande C , Denicola A . Potential modulation of Sirtuins by oxidative stress. Oxidative Med Cell Longev. 2016;2016:9831825. 10.1155/2016/9831825 PMC4691645 26788256

[121] Rubin LJ , Magliola L , Feng X , Jones AW , Hale CC . Metabolic activation of AMP kinase in vascular smooth muscle. J Appl Physiol. 2005;98(1):296–306. 10.1152/japplphysiol.00075.2004 15377643

[122] Pattingre S , Tassa A , Qu X , Garuti R , Liang XH , Mizushima N , et al. Bcl‐2 antiapoptotic proteins inhibit Beclin 1‐dependent autophagy. Cell. 2005;122(6):927–939. 10.1016/j.cell.2005.07.002 16179260

